# Hepatic stereological analysis in obese Zucker rats (Leprfa) with dyslipidemia

**DOI:** 10.1590/acb402325

**Published:** 2025-03-14

**Authors:** Silvio Pires Gomes, Gabriela Salim de Castro, Vinicius Pedro Silva de Oliveira, Bruno Cogliati, Andressa Galvão da Silva Iacopino, Ivanir Santana de Oliveira Pires, Bruno Cesar Schimming, Fernanda Gosuen Gonçalves Dias, José Roberto Kfoury, Tais Harumi de Castro Sasahara

**Affiliations:** 1 Universidade de São Paulo – Faculdade de Medicina Veterinária e Zootecnia – Departamento de Cirurgia – São Paulo (SP) – Brazil.; 2 Universidade de São Paulo – Faculdade de Medicina – Departamento de Cirurgia – São Paulo (SP) – Brazil.; 3 Centro Universitário Braz Cubas – Faculdade de Medicina Veterinária – Mogi das Cruzes (SP) – Brazil.; 4 Universidade de São Paulo – Faculdade de Medicina Veterinária e Zootecnia – Departamento de Patologia – São Paulo (SP) – Brazil.; 5 Universidade Nove de Julho – Faculdade de Medicina Veterinária – São Paulo (SP) – Brazil.; 6 Universidade de São Paulo – Instituto de Ciências Biomédicas – Departamento de Cirurgia – São Paulo (SP) – Brazil.; 7 Universidade Estadual Paulista – Instituto de Biociências – Departamento de Biologia Estrutural e Funcional – Botucatu (SP) – Brazil.; 8 Universidade de Franca – Faculdade de Medicina Veterinária – Departamento de Ciência Animal – Franca (SP) – Brazil.

**Keywords:** Obesity, Rats, Zucker, Liver, Fatty Liver, Hepatocytes

## Abstract

**Purpose:**

To characterize histologically and stereologically the hepatic steatosis in obese Zucker (fat, n = 6, with a mutation in the leptin receptor – Leprfa) and control Zucker (lean, n = 6) rats, analyzing macroscopic and microscopic differences to understand the influence of obesity on hepatic pathology.

**Methods:**

Zucker rats were fed standard chow for 90 days. Macroscopic, qualitative, and histoquantitative (stereological) approaches were used, involving body and liver weight measurement, morphological analysis, and histopathological classification of metabolic dysfunction-associated steatotic liver disease.

**Results:**

Zucker fat rats had higher body weight (*p* = 0.0022), liver weight (*p* = 0.0022), serum total cholesterol (*p* = 0.0022), and triacylglycerol (*p* = 0.0022) compared to Zucker lean rats. Stereological analysis showed that hepatocyte volume density (*p* = 0.0022) and total hepatocyte volume (*p* = 0.0001) were lower, and the volume density (*p* = 0.002) and total volume of steatosis (*p* = 0.002) were higher in Zucker fat rats compared to lean rats.

**Conclusion:**

The findings indicated that obesity induces significant alterations in the hepatic morphology of Zucker rats, showing that hepatocyte volume is lower in obese animals. This study reinforces the utility of the obese Zucker rat model to investigate the effects of obesity on liver health and suggests hepatic steatosis requires therapeutic strategies focused on modulating these parameters.

## Introduction

Obesity is globally escalating, transcending economic and social barriers, and is classified as a chronic non-communicable disease leading to health issues like metabolic dysfunction-associated steatotic liver disease (MASLD). The prevalence of obesity is rising in both developed and developing countries, emphasizing it as a public health concern^1^. Additionally, studies indicate that conditions like type-II diabetes and metabolic syndrome, associated with obesity, hasten the development of cardiovascular diseases^2^.

Knowing the pathogenic mechanisms of obesity is crucial for developing research and intervention methods. The importance of animal models in medical research to study metabolism and the pathogenesis of obesity-related diseases is highlighted, noting that results from animal models are not always directly transferable to humans, underscoring the need for valid models for studies^3^^,^ 4. Obesity is not merely an issue of excess weight but also an inflammatory state affecting vital organ functions, such as the liver. Hepatic steatosis, a component of MASLD, is considered a direct manifestation of obesity’s metabolic impact on the liver. The link between obesity, insulin resistance, and hepatic dysfunction is well documented, with studies showing a significant correlation between hepatic fat accumulation and the development of insulin resistance^5^.

The value of obese Zucker rats as a model for studying obesity and its metabolic complications, including liver alterations, is noteworthy. Characterizing these animals, which exhibit spontaneous obesity, hyperphagia, glucose intolerance, and dyslipidemia, provides valuable insights into the pathophysiological mechanisms underlying obesity and its systemic consequences^6^^,^ 7.

Understanding the changes in the microvascular and tissue alterations in the liver are fundamental to comprehend the progression of hepatic steatosis and the transition to more severe liver disease forms. The obese Zucker rats serve as a vital model for studying obesity and its outcomes, including steatosis and hepatic microvascular changes, aiding in elucidating the complex metabolic and pathophysiological interactions linked to obesity and its hepatic complications^8^^–^ 10.

Rosenstengel et al.^11^ demonstrated that high-fat diets induce hepatic steatosis, highlighting the role of Zucker rats in researching liver diseases associated with obesity. Choosing an appropriate animal model, such as the obese Zucker rats, is crucial for mimicking human obesity and its repercussions, facilitating the identification of mechanisms of obesity-related diseases, and developing therapeutic interventions^3^^,^ 4.

There is an urgent need to address the global obesity epidemic and its health consequences, emphasizing the importance of studies exploring the complex mechanisms of obesity and its relationship with liver health. Utilizing animal models, like the Zucker rats, represents a crucial platform for such investigations, aiding in the development of specific therapeutic approaches and enhancing the understanding of liver pathologies linked to obesity. Therefore, this study aimed to characterize the hepatic stereological alterations related to obesity in a widespread animal model.

## Methods

### Animals

The present study was conducted in accordance with the ethical principles of animal experimentation of the Ethics Committee (CEUA) of the Faculdade de Medicina Veterinária e Zootecnia da Universidade de São Paulo (FMVZ/USP), under the number CEUA/FMVZ/USP: 2868291121. For this research, 12 male Zucker rats, 90 days old, were used. They were separated in two groups:

- Fat group: six animals with a mutation in the leptin receptor (Leprfa);
- Lean group: six animals without the mutation.

They were obtained from the Center for the Development of Experimental Models for Biology and Medicine of the Universidade Federal de São Paulo and kept for 24 hours at the FMVZ/USP until the euthanasia. The experimental procedures and euthanasia were carried out in accordance with the standards set by the National Council for the Control of Animal Experimentation. No obesity induction procedure was employed, as the Zucker obese rat harbors a spontaneous autosomal mutation in the leptin receptor gene and develops a hyperphagic phenotype leading to morbid obesity, glucose intolerance, and insulin resistance^6^. Zucker obese rats show disturbance in body energy expenditure starting at seven days after birth^12^.

### Sample collection for biochemical analysis

Animals were fasted for 12 hours before blood collection and euthanasia. The process of blood collection for biochemical profile analysis followed the protocol described by Lee and Goosens^13^. The animals’ tails were immersed in water at 42°C for 40 to 50 seconds and then aseptically treated with 0.5% chlorhexidine digluconate (Riohex, Rioquimica LTDA Pharmaceutical Industry). Subsequently, venipuncture of the caudal vein was performed using a catheter attached to a 3-mL syringe. The collected blood was stored in Vacutainer tubes (SST Gel and clot activator, Becton Dickinson), centrifuged at 3,000 rpm for 15 minutes at 4°C, and the serum was used for the measurement of triglycerides (mg/dL) and total cholesterol (mg/dL). The serum samples were processed on an automated biochemical analyzer (Chemwell model), using commercial kits (Triglycerides Liquiform kit and the Cholesterol Liquiform Labtest Diagnóstica, Brazil), both by the enzymatic-trinder method.

### Euthanasia

The animals were exposed to anesthetic gas (5% isoflurane). For euthanasia, the anesthetic was diluted in oxygen through a vaporizer and introduced into the chamber at the highest possible concentration. Additionally, a gauze soaked with anesthetic was placed in a closed container along with the animal so that it was only in contact with the anesthetic vapor. Subsequently, an initial perfusion in the heart with phosphate-buffered saline (PBS) was conducted to wash the vascular system. This was followed by perfusion with 4% paraformaldehyde in PBS. The livers were then removed from the animals and weighed before being immersed in the same fixative solution and stored at a temperature of 4°C.

### Qualitative study

#### Morphological characterization of the liver

Deparaffinization and rehydration were performed by placing the slides in an oven at 56°C for 1 hour, and then passing them through a series of xylene and decreasing concentrations of alcohols, respectively. Staining was done for 15 seconds in filtered hematoxylin, followed by washing in water, and then for 4 minutes in filtered eosin, differentiated in 70% alcohol, with the intensity controlled under a microscope. Dehydration was carried out in a series of increasing alcohols and xylene, followed by mounting of the slides with permount (Fisher Scientific, Fisher Chemalert Guide, New Jersey, United States of America) on a slide and cover slip. The slides were evaluated, and images captured using a digital camera (Nikon DS-Ri1 model) attached to a light microscope (Nikon Eclipse 80i) and the capture program software (NIS-Elements F3.2).

#### Histopathological classification of MASLD

The histopathological assessment was based on the standardized classification by the metabolic dysfunction-associated steatohepatitis (MASH) clinical research network, which designated and validated a scoring system for MASLD activity in clinical studies, the MASLD Activity Score^14^. The score for each animal was determined by the sum of the grades of macro/microvesicular steatosis (0 to 3), evaluated on the slides stained with hematoxylin and eosin.

#### Stereological study

**Figure 1:**
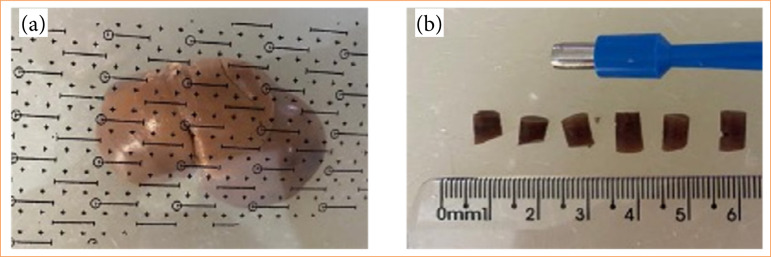
Representative liver and sample collection from a control animal. **(a)** Point system used with “frame” to collect fragments from the liver lobes of animals from both groups; **(b)** 4-mm circular “Punch” scalpel, used for liver biopsy fragment retrieval.

Source: Elaborated by the authors.

Liver biopsies were collected systematically and randomly using a 4-mm “Punch” circular frame and scalpel (Uniqmed), ensuring representativeness and uniformity of the liver tissue for histological analyses. This procedure, consistently applied at predetermined points of the organ, allowed for the collection of 16 fragments from the hepatic lobes of both Zucker lean and fat groups. The employed methodology ensured the standardization of sample collection, minimizing bias and enhancing the reliability of the histopathological results^15^, as illustrated in Fig. 1.

### Morphoquantitative (stereological) study of the liver

#### Liver volume

The liver volume was estimated based on Archimedes’ principle through the formula for irregular body density, dividing mass (weight in grams) by volume (displacement in water, in mL). For calculations related to hepatic volume, the 1.06-g/cm^3^ density of liver tissue was used, as established by Gomes et al^15^., by Eq. 1:


V=M/D (1)

where: V: liver volume; M: mass (weight in grams of the liver); D: liver density.

The estimated liver volume result is expressed in cm^3^.

#### Volume density

The volume density in the liver refers to the fraction occupied by hepatocytes and fat in Zucker lean and fat rats. A system of random and systematic points is used to calculate this density. Volume is expressed by the ratio of points touching hepatocytes (P(h)) and lipid droplets (P(ld)) to the total reference space (P(rs)). Thus, the volume density (Vv) is given by Eq. 2, to calculate hepatocyte volume density, and Eq. 3, to calculate lipid droplets volume density:


Vv(h,rs)=ΣP(h)/ΣP(rs) (2)

Vv (ld,rs)=ΣP(ld)/ΣP(rs) (3)

**Figure 2:**
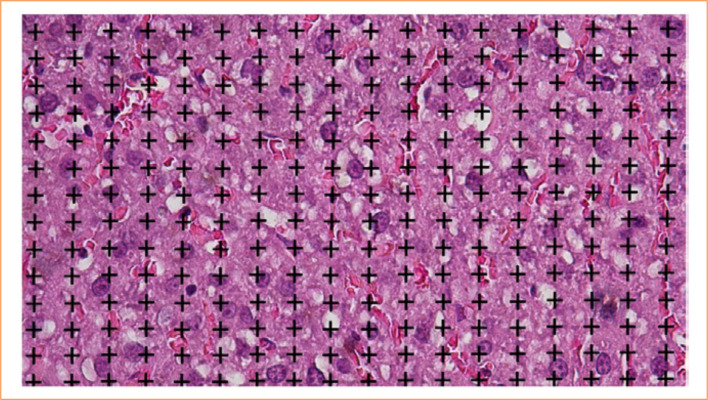
Digital illustration of the test system, consisting of random and systematic points, used to estimate the volume density of hepatocytes and lipid droplets. Scale bar: 20 μm.

Source: Elaborated by the authors.

These values are represented in percentage, providing a quantitative measure of hepatic alterations^15^^–^ 17, as shown in Fig. 2.

#### Estimation of total volume of hepatocytes and steatoses

The estimation of the total volume of hepatocytes and lipid droplets involves the application of stereological formulas to quantify these components in the liver^15^. The total volume (V) of hepatocytes or fat is calculated by multiplying the volume density (Vv) by the reference volume (Vref) of the liver. The volume density (Vv) is determined by the ratio of the number of points intercepting hepatocytes or lipid droplets (Σp) to the total number of points in the reference space (ΣP(rs)). Therefore, the formula to calculate the total volume of hepatocytes (V(hep)) or lipid droplets (V(ld)) is Eq. 4:


V(hep)=Vv(liver)×Vhep (4)

Moreover, the formula to calculate the total volume of fat is Eq. 5:


V(ld)=Vv(liver)×Vhep (5)

### Statistical analysis

The distribution of variables was checked using the Shapiro-Wilk’s test. Parametrically distributed variables are presented with mean and standard deviation and were compared using the Student’s t test. Non-parametric variables are presented with median and interquartile range and were compared using the Mann-Whitney’s test. Spearman’s correlation test was used for assessing the correlation of non-parametric variables. Results were considered significant when *p* < 0.05. Statistical analysis was performed using GraphPad Prism 8.4.3 software.

## Results

### Body and liver weight

**Figure 3:**
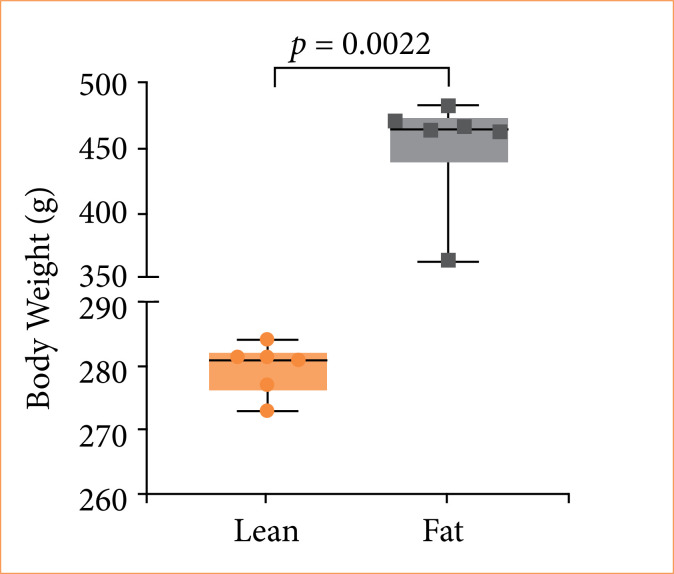
The measurement of body weights in Zucker lean and Zucker fat rats indicated a significant difference (*p* = 0.0022). In the graphs, squares and circles represent individual values, while horizontal bars display the group medians.

Source: Elaborated by the authors.

The body weight of Zucker rats is illustrated in Fig. 3. It was observed that animals in the fat group exhibited significantly higher body weights compared to those in the lean group.

**Figure 4:**
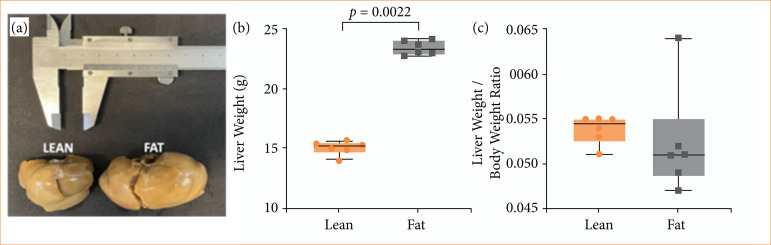
**(a)** Comparative macrographic photograph of Zucker rats’ livers; **(b)** the weights of Zucker lean and fat rats’ livers showed a significant difference (p = 0.0022); **(c)** the body weight and liver weight ratio in Zucker lean and Zucker fat rat groups. In this analysis, circles and squares represent individual values, while horizontal bars display the medians of each group.

Source: Elaborated by the authors..

The liver weight of obese Zucker rats was higher than the weight of livers from the lean group, as shown in Fig. 4. The ratio of liver weight to total body weight did not differ between the groups.

### Liver microstructure

**Figure 5:**
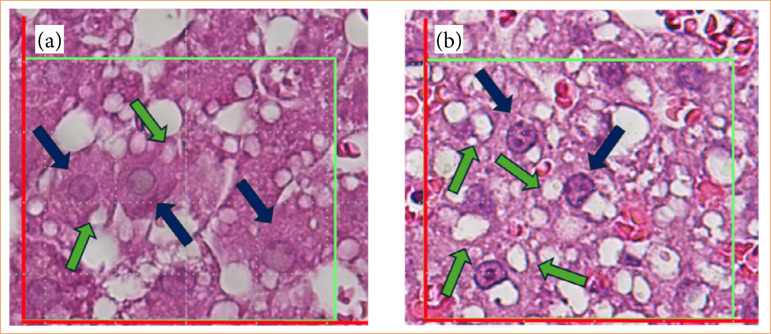
Comparative photomicrograph of hepatic microstructure. **(a)** The liver of Zucker lean; **(b)** livers from Zucker fat rats. The blue arrows point to hepatocytes, highlighting their smaller size in **(a)** compared to **(b)**, while the green arrows indicate steatosis, with lipid droplets being smaller in **(a)** than in **(b)**. Staining: hematoxylin-eosin. Scale: 50 μm.

Source: Elaborated by the authors.

Histologically, the hepatic lobule presents a hexagonal-like shape, with a central lobular vein and portal triads at the end of this hexagon. The columns of hepatocytes are arranged side by side, forming the hepatocellular trabecula, and between them are located the hepatic sinusoids, which transport blood arriving through the branches of the portal vein and hepatic artery to the central vein. The liver structures (distribution of hepatocytes) were not modified in the evaluated groups, as shown in Fig. 5. However, it is possible to note that the number and size of lipid droplets were higher in the fat group compared to the lean group.

### Histopathological classification of hepatic steatosis

**Figure 6:**
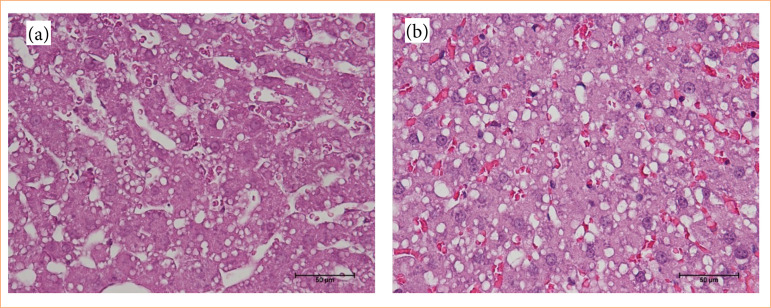
Photomicrograph of Zucker fat rats’ hepatocytes. **(a)** Photomicrograph of the liver from a Zucker lean rat highlighting smaller and fewer lipid droplets; **(b)** photomicrograph of the liver from a Zucker fat rat displaying larger lipid droplets. Staining performed with hematoxylin-eosin. Scale bar: 50 μm.

Source: Elaborated by the authors.

The liver biopsies from lean and fat groups were examined by microscopic analysis after staining with hematoxylin-eosin, aiming to classify histopathological changes and evaluate the presence of MASLD. This detailed examination revealed structural differences, including fat accumulation, inflammation, and tissue damage, especially notable in the fat group, in which larger lipid droplets were observed. In Fig. 6, it is possible to significantly observe alterations in the liver of rats from the fat group, with the presence of a higher number and size of lipid droplets, indicating a severe hepatic steatosis.

**Figure 7:**
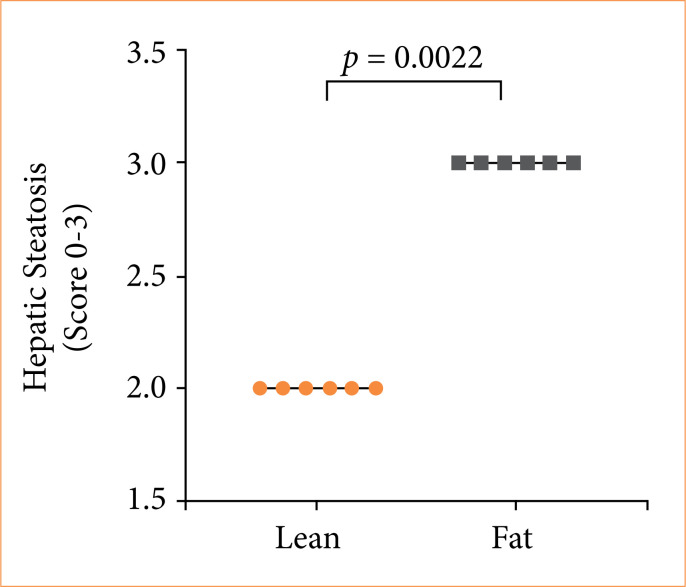
The result of the steatosis degree assessment, ranging from 0 to 3 (*p* = 0.0022), where 0 indicates absence of steatosis and 3 represents severe steatosis. The distribution of grades illustrates the prevalence and intensity of hepatic steatosis in the studied groups, providing a quantitative view of the liver disease burden. Circles and squares indicate individual values, while horizontal bars display group medians.

Source: Elaborated by the authors.

The degree of hepatic steatosis was higher in the fat group, as showed in Fig. 7. Both groups showed microvesicular steatosis. Even the lean group presented some degree of hepatic lipid accumulation.

### Quantitative stereological study

**Figure 8:**
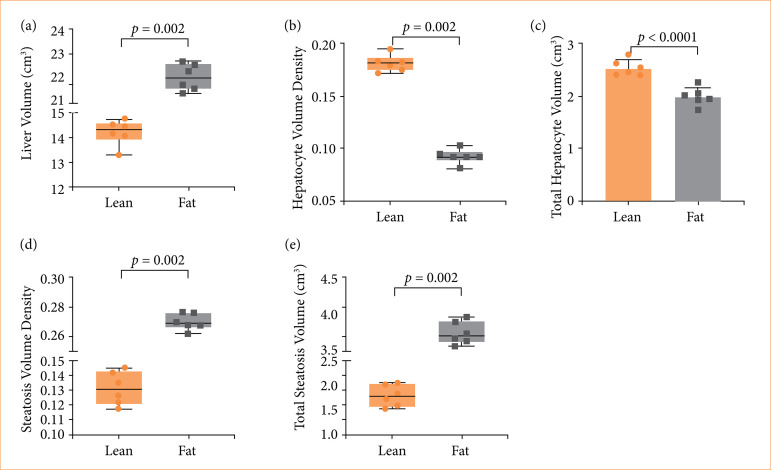
Liver parameter of Zucker lean and fat rats. **(a)** The liver volumes, estimated by the Archimedes’ principle (*p* = 0.002); **(b)** the hepatocyte volume density (*p* = 0.002); **(c)** the total volume of hepatocytes (*p* = 0.001); **(d)** the volume density of steatosis (*p* = 0.002); **(e)** the total volume of steatosis (*p* = 0.002) of the lean and fat groups. Circles and squares are representing individual values and horizontal bars the group averages.

Source: Elaborated by the authors.

Hepatocyte volume density provides a crucial assessment of liver condition, indicating liver health and functionality. Normal values indicate adequate liver function, while alterations may suggest pathologies such as cellular degeneration or hypertrophy, making this measure essential for the diagnosis and monitoring of liver diseases. The liver volume of the rats from the fat group was higher, while the hepatocyte volume density and total hepatocyte volume were lower compared to the lean group, as shown in Fig. 8. The volume density of steatosis (Vv) and the total volume of steatosis (Vesteatose) in the animals’ livers was higher in Zucker fat compared to Zucker lean (*p* = 0.002).

### Biochemical assessments

**Figure 9:**
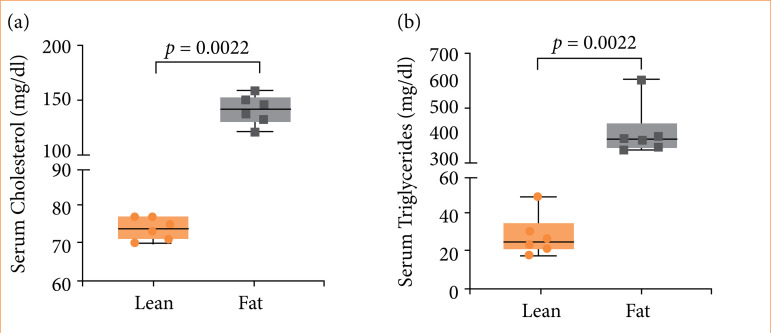
Serum total cholesterol and triglycerides of Zucker lean and fat rats. **(a)** Levels of total cholesterol in serum (mg/dL) (*p* = 0.0022). **(b)** Serum concentrations of triglycerides (mg/dL) (*p* = 0.0022) of Zucker lean and Zucker fat rat groups. Circles and squares symbolize individual values, while horizontal bars represent group medians. The significant statistical difference between the groups highlights the metabolic variations associated with different genetic profiles of rats.

Source: Elaborated by the authors.

The serum total cholesterol and triglycerides concentrations showed differences between lean and fat groups, as illustrated in Fig. 9.

### Correlations

**Figure 10:**
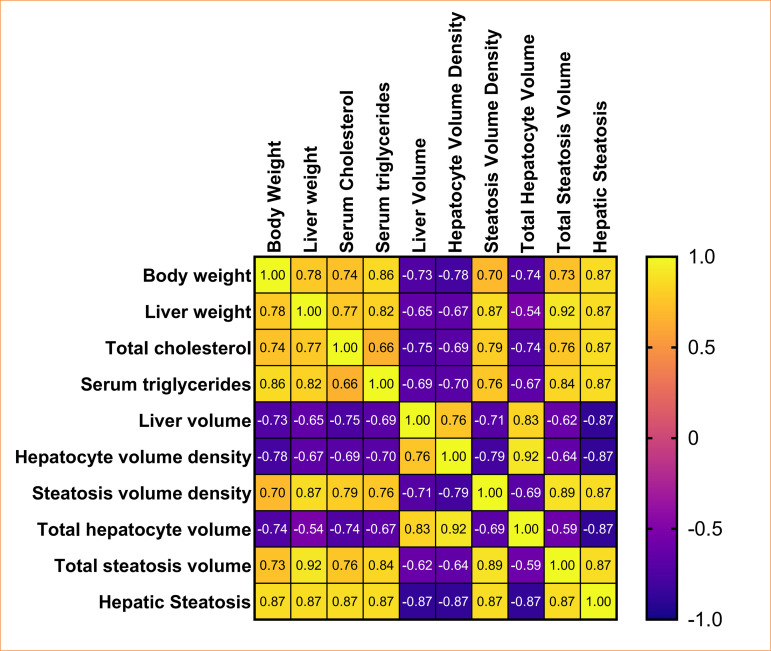
Correlations of variables compared with Spearman’s test. The graph correlates all the data collected and analyzed during the histological and stereological characterization of hepatic steatosis in Zucker rats.

Source: Elaborated by the authors.

The correlations analysis showed that hepatic stereological parameters were associated with body weight, liver weight, serum total cholesterol, and serum triglycerides, as showed in Fig. 10. Liver volume (*p* = 0.009; *p* = 0.026; *p* = 0.007; *p* = 0.0155), hepatocyte volume density (*p* = 0.004; *p* = 0.020; *p* = 0.0015; *p* = 0.0144), and total hepatocyte volume (*p* = 0.008; *p* = 0.076; *p* = 0.008; *p* = 0.0203) were negatively correlated with body weight, liver weight, serum total cholesterol, and serum triglycerides. Steatosis volume density (*p* = 0.014; *p* = 0.0004; *p* = 0.003; *p* = 0.005), total steatosis volume (*p* = 0.009; *p* = 0.0001; *p* = 0.006; *p* = 0.0011), and hepatic steatosis (*p* = 0.002; *p* = 0.0021; *p* = 0.002; *p* = 0.002) were positively associated with body weight, liver weight, serum total cholesterol, and serum triglycerides.

## Discussion

The World Health Organization^18^ defines obesity as an excessive accumulation of fat that poses a health threat, highlighting it as a key factor in global chronic diseases. Associated with conditions such as cardiovascular diseases, type-2 diabetes, hypertension, certain cancers, and MASLD, obesity arises from genetic, dietary, sedentary, and environmental factors. The study focused on the histological and stereological characterization of hepatic steatosis in Zucker rats, aiming to deeply characterize the liver alterations in this important animal model of obesity. Obese Zucker rats showed a higher incidence and severity of hepatic steatosis, underlining the connection between obesity and hepatic complications. Moreover, the obese rats presented lower hepatocyte volume density, which was negatively associated with liver weight, serum total cholesterol, and serum triacylglycerols.

Rats fed a high-fat diet presented higher liver fibrosis, which can modify the structure of hepatocytes^19^. Tong et al.^20^ observed that, while Zucker lean rats exhibited minimal increases in triglycerides and cholesterol on high-fat diets, Zucker fat rats displayed elevated levels of these lipids, reflecting a higher risk of hepatic steatosis and dyslipidemia, as well as increased susceptibility to liver damage, as evidenced by alanine transaminase concentration. The study emphasizes the importance of weight management in preventing and treating hepatic steatosis.

In a normal liver, the hepatic structure comprises lobules with hepatocytes organized in plates, separated by sinusoidal spaces and supported by reticular fibers^20^. The centrolobular vein facilitates blood flow without a muscular layer, lined by endothelial cells. In Zucker fat rats, changes such as excessive accumulation of fat in hepatocytes occur, affecting hepatic architecture and function. These differences are crucial for understanding the impact of obesity and altered lipid metabolism on liver health, elucidating the mechanisms of MASLD. Despite the similar anatomical basis between Zucker lean and fat rats, fat accumulation is more pronounced in Zucker fat, indicating hepatic steatosis. Liang et al.^21^ developed a scoring system for MASLD in rodents, noting that ballooning, a sign of hepatocellular injury, is less common in rodents compared to humans, highlighting differences between experimental models and human pathology.

MASLD represents a spectrum of liver pathologies ranging from MASH, with a risk of progression to fibrosis, cirrhosis, and even hepatocellular carcinoma^21^. In steatosis, the liver accumulates fat, differentiating into macrovesicular, with large fat droplets displacing the cell nucleus, and microvesicular, with small droplets dispersed in the cytoplasm. In Zucker rats, especially obese ones (Zucker fat), macrovesicular steatosis is common due to metabolic disorders. Although less frequent, microvesicular steatosis occurs in conditions of severe metabolic stress or exposure to toxins. Studying these conditions in Zucker rats clarifies the progression and mechanisms of obesity-related liver disease, contributing to the development of therapies against serious complications such as MASH^22^^,^ 23.

It has been demonstrated that obese Zucker rats revealed elevated levels of enzymes indicative of hepatic injury, suggesting that hepatic steatosis in Zucker fat rats may resemble the pathological condition of MASLD in humans, although ballooning is less prominent^21^. Hepatic steatosis in Zucker fat rats is associated with an increased risk of advanced liver injury, but, as per Liang et al.^21^, ballooning is less prevalent in rodents than in humans, indicating differences in disease manifestation. Stereology helps quantify pathological changes, such as hepatic steatosis, in Zucker rats, while Tong et al.^20^ provide essential anatomical details of the rat liver, which is important for understanding the normal hepatic structure and observed pathological changes.

LaBranche et al.^24^ investigated hepatic steatosis in lean Zucker rats fed a high-fat diet, linking it to asymptomatic metabolic syndrome and increased sensitivity to drug-induced liver damage, highlighting the relevance of lean Zucker rats as a model for hepatic toxicity studies in contexts of altered metabolism. These authors reported that Zucker lean rats fed a high-fat diet develop mild hepatic steatosis and compensatory hyperinsulinemia but exhibit increased susceptibility to hepatocellular damage after exposure to acetaminophen, presenting elevated levels of liver injury and necrosis enzymes. This is related to the accumulation of acetaminophen in the liver and changes in MDR1 protein, suggesting that diet-induced hepatic steatosis makes lean Zucker rats more sensitive to drug-induced liver injuries. This study underscores the importance of considering mild steatosis as a risk factor for liver damage. Sheng et al.^25^ observed that lean Zucker rats maintain a stable hepatic lipid and enzymatic profile under a high-fat diet, demonstrating metabolic resilience, while obese Zucker rats exhibit high levels of lipids and liver enzymes, indicating a predisposition to metabolic disorders and increased vulnerability to liver damage.

Sheng et al.^25^ emphasized the utility of lean and obese Zucker models to study the connections between obesity, lipid metabolism, and liver health. We observed a direct correlation between body weight and levels of cholesterol and triglycerides, with significant increases in the fat group. Comparatively, Schonfeld et al.^26^ and Wang et al.^27^ noted elevated lipid profiles in Zucker fat rats, related to increased hepatic production of lipoproteins. Csonka et al.^28^ also reported lipid accumulations and liver damage in rats with high cholesterol diets, resembling findings in Zucker fat rats. Shmarakov et al.^29^ highlighted the vital role of retinoids in liver regeneration, showing that their depletion compromises post-hepatectomy liver recovery, underlining the complexity of interactions between metabolism, liver damage, and regeneration.

The depletion of retinoids, which affects liver regeneration in mice, can be compared to the metabolic alterations in obese Zucker rats, such as hepatic steatosis, impacting the liver’s regenerative capacity. This parallel suggests that both retinoids and metabolic conditions influence liver regeneration. Vekic et al.^30^ found structural hepatic changes induced by hypervitaminosis A in rats, including vacuolation and fibrosis, like the changes in obese Zucker rats, in which steatosis is marked by hepatocyte vacuolation due to lipid accumulation. Kucera et al.^31^ discussed the importance of experimental rat models for studying MASLD, highlighting the need for models that accurately reflect human pathology, given the disease’s complexity.

Stereology, applied to the liver, assesses structural and quantitative changes, as in hepatic steatosis of lean and fat groups. This technique allows for identifying changes in volume and number of hepatocytes and in fat accumulation, essential for correlating dietary or genetic impacts with liver health. Rothman et al.^32^ validated a stereological method to accurately estimate the size and density of particles in tridimensional (3D) from bidimensional images, addressing the challenge of “lost caps”. Veras et al.^33^ discussed the use of stereology for 3D quantitative analysis in microscopy, applying principles like the Cavalieri method for volumetric estimates and point counting for volume fractions, as detailed by Gomes et al.^15^. Additional methods include line intersection for surface area and length, and the disector for numerical cell density, facilitating unbiased analyses of tissue structures.

The study by Gomes et al.^15^ on hepatic recovery after protein refeeding in Zucker rats showed that protein reintroduction increases the number and size of hepatocytes, reversing hepatic atrophy. The research highlights the liver’s resilience and regenerative capacity in response to nutritional changes, in which protein malnutrition reduced liver volume and altered cell morphology, while refeeding partially restored these structures. Santos et al.^34^ and Rees et al.^35^ were cited to support the persistence of hepatic changes despite refeeding. Abrahão et al.^36^ emphasized stereology for quantitative analyses in biological development, while Gomes et al.^37^ applied it to assess the impacts of protein deficiency on neurons, demonstrating the utility of stereology in understanding morphological and functional changes in biological tissues.

## Conclusion

Stereological and morphological analysis revealed significant hepatic steatosis in obese Zucker rats. This methodology provided a detailed assessment of hepatic changes in this animal model of obesity. The results presented here showed for the first time that Zucker obese rats present lower hepatocyte volume density compared to lean counterparts. These findings highlight the need for future research to unravel the mechanisms of these conditions and to develop more effective treatments for obesity-linked liver diseases. Stereology has proven crucial in quantifying hepatocytes and lipid droplets, underpinning advanced studies and the creation of specific therapeutic strategies.

## Data Availability

All data are available in the article.
